# Beverage Consumption and Ulcerative Colitis: A Case-Control Study from Saudi Arabia

**DOI:** 10.3390/ijerph19042287

**Published:** 2022-02-17

**Authors:** Anas Almofarreh, Haytham A. Sheerah, Ahmed Arafa, Shaik Shaffi Ahamed, Osama Alzeer, Weiam Al-Hunaishi, Mohamed Ma Mhimed, Ali Al-Hazmi, Sin How Lim

**Affiliations:** 1 Department of Social and Preventive Medicine, Faculty of Medicine, University of Malaya, Kuala Lumpur 50603, Malaysia; aalmofarreh@moh.gov.sa (A.A.); waam59@hotmail.com (W.A.-H.); howie.ceria@gmail.com (S.H.L.); 2 Ministry of Health, Riyadh 15595, Saudi Arabia; 3 Health Promotion and Health Education Research Chair, King Saud University, Riyadh 11451, Saudi Arabia; oalzeer@hotmail.com (O.A.); aalhazmii@ksu.edu.sa (A.A.-H.); 4 Public Health, Department of Social Medicine, Graduate School of Medicine, Osaka University, Suita 565-0871, Japan; ahmed011172@med.bsu.edu.eg; 5 Department of Preventive Cardiology, National Cerebral and Cardiovascular Center, Suita 564-8565, Japan; 6 Department of Public Health, Faculty of Medicine, Beni-Suef University, Beni-Suef 62521, Egypt; 7 Department of Family and Community Medicine, College of Medicine, King Saud University, Riyadh 11451, Saudi Arabia; sshaik@ksu.edu.sa; 8 Department of Clinical Nutrition, King Khalid University Hospital, King Saud University, Riyadh 11451, Saudi Arabia; 9 Cell and Tissue Culture Department, Libyan Center for Biotechnology Research, Tripoli 30313, Libya; mkhalifa2020@yahoo.com; 10 Head of Information, Research and Data Analysis Department, National Center for Disease Control (NCDC)—Aljafara Branch, Tripoli 71171, Libya; 11 Scientific Research Unit, Research and Development Department, Primary Health Care Institute, Tripoli 00218, Libya; 12 Centre of Excellence for Research in AIDS (CERiA), Faculty of Medicine, University of Malaya, Kuala Lumpur 50603, Malaysia

**Keywords:** ulcerative colitis, tea, coffee, carbonated soft drinks, case-control study, Saudi Arabia

## Abstract

Background: The association between beverage intake and ulcerative colitis (UC) is not well-established, with no available data from Arab countries. Herein, we investigated the potential association of consuming coffee, tea, and carbonated soft drinks with UC among a population from Saudi Arabia. Methods: This hospital-based case-control study used data of 171 newly diagnosed UC patients and 400 patients with other gastrointestinal conditions who served as controls. All UC cases were ascertained by endoscopy, while beverage intake was assessed by a questionnaire that was completed before diagnosis. We computed odds ratios (ORs) and 95% confidence intervals (95% CIs) of UC and UC extension for frequent versus infrequent intakes of coffee, tea, and carbonated soft drinks using logistic regression. Results: Overall, 23.4% of UC patients had pancolitis, 21.1% extensive, 51.4% left-sided, and 4.1% proctitis. UC patients had a similar sex distribution to the controls but were older and had a lower BMI. After adjustment for age, sex, body mass index, and smoking history, frequent intakes of coffee and tea were associated with lower odds of UC: 0.62 (0.42, 0.91) and 0.53 (0.35, 0.79), respectively. On the other hand, frequent intakes of carbonated soft drinks were associated with increased odds of UC: 9.82 (6.12, 15.76). The frequency of beverage consumption was not associated with UC extension. Conclusion: UC was negatively associated with frequent coffee and tea consumption but positively associated with frequent carbonated soft drink intake in Saudi people. More population-based prospective cohort studies are needed to confirm our findings.

## 1. Introduction

Ulcerative colitis (UC) is a chronic inflammatory disease starting in the rectum and extending to proximal segments of the colon [1,2]. It is clinically characterized by frequent relapses of bloody diarrhea, abdominal pain, and bloating [1,2] that lead to poor quality of life and heavy financial burden [3,4]. The incidence and prevalence of UC have been increasing steadily over the past decades worldwide [5].

The real etiology of UC is still unknown; however, several genetic, environmental, and nutritional risk factors have been indicated, such as family history of UC, smoking, and some medications, including hormone replacement therapy, oral contraceptives, and non-steroidal anti-inflammatory drugs [1]. Among these factors, a growing body of evidence, however inconclusive, has suggested that beverage consumption, namely coffee, tea, and soft drinks, could be related to UC risk [6]. However, the epidemiological studies reporting this evidence were performed in Western and East-Asian communities and had inconsistent findings [7,8,9,10,11,12,13]. In addition, the association between beverage consumption and UC extension has never been studied.

Saudi Arabia is among the largest countries in the East Mediterranean Region and is showing an increasing incidence of UC [14,15]. The Saudi population has unique dietary behaviors that are significantly different from those of Western and East-Asian populations. For example, the literature has shown a high prevalence of beverage consumption among Saudi people [16,17,18]. 

We therefore conducted this case-control study using data of newly diagnosed UC patients from a large polyclinic in Saudi Arabia to investigate the possible relationship between the intakes of coffee, tea, and carbonated soft drinks and UC or UC extension. We aspired that the results of this study could give us a better understanding of the nutritional risk factors for UC among Saudi patients.

## 2. Methods

### 2.1. Study Population and Setting

This hospital-based case-control study used secondary data of 171 patients with UC and 400 patients with other gastrointestinal conditions who were diagnosed between January 2009 and December 2017 in one private polyclinic in Riyadh, the Capital of Saudi Arabia. Our eligibility criteria for UC patients included (1) newly diagnosed patients, (2) UC diagnosis was ascertained by lower endoscopy with biopsies, (3) >18 years, (4) Saudi citizens, and (5) agreed to participate in the study by giving informed consent. Patients who served as controls had no confirmed or suspected inflammatory bowel disease, malignancy, diverticulosis, or polyposis based on their manifestations and laboratory findings. 

### 2.2. Ulcerative Colitis Diagnosis

All patients with UC manifestations, such as abdominal pain, diarrhea, bloating, loss of appetite, unexplained weight loss, or bloody stool, had laboratory investigations, including urine and stool analysis for biomarkers of inflammation and serum complete blood count, C-reactive protein, erythrocyte sedimentation rates, bilirubin, alanine aminotransferase, creatinine, and alkaline phosphatase. Patients with probable UC manifestations and laboratory findings had lower gastrointestinal endoscopy with high-definition endoscopes utilizing either Olympus, Pentax, or Fujinon video scopes, and their biopsies were assessed histopathologically.

### 2.3. Beverage Intake Assessment

The intakes of coffee, tea, carbonated soft drinks, and alcohol were assessed during the first visit, before undergoing any laboratory or endoscopic intervention, using a self-administered questionnaire. The questions on beverage intakes were as follows: “*How frequently do you drink coffee?*”, “*How frequently do you drink tea?*”, “*How frequently do you drink carbonated soft drinks?*”, and “*How frequently do you drink alcohol?*”. The available responses were “*never or rarely*”, “*once/week*”, “*twice/week*”, and “*daily*”. Tea included all kinds of tea, such as black and green tea, while carbonated soft drinks included sweetened and non-sweetened carbonated soft drinks. 

### 2.4. Covariates

Age (years), sex (men or women), weight in kg, and height in cm, body mass index (BMI; calculated by weight in kg/height in m^2^), history of smoking (yes or no), and medical history of cardiovascular diseases (yes or no) and neurological disorders (yes or no) were assessed by the same baseline questionnaire. Illiterate participants sought help from investigators.

### 2.5. Statistical Analyses

The Chi-squared test and *t*-test were used to detect the differences in proportions (sex and smoking history) and mean values (age and BMI) between cases and controls. To obtain statistical power, the responses “*never or rarely*”, “*once/week*”, “*twice/week*” were merged into one category “*infrequent*”, while the daily intake was considered “*frequent*”. Since only three subjects reported alcohol intake, this question was omitted. The odds ratios (ORs) with their 95% confidence intervals (95% CIs) of UC versus controls for frequent versus infrequent intakes of coffee, tea, and carbonated soft drinks were calculated separately by logistic regression. Three regression models were presented: model I: unadjusted, model II: adjusted for age and sex, and model III: adjusted for age, sex, BMI, and smoking history. Since all UC patients and controls had a negative history of cardiovascular and neurological diseases, these variables were not included in the regression models. Then, we calculated using the same regression models the ORs (95% CIs) of severe forms of UC (pancolitis and extensive) versus other forms of UC (left-sided and proctitis) for frequent versus infrequent intakes of coffee, tea, and carbonated soft drinks. Data were analyzed using the Statistical Package for Social Science (SPSS) released in 2013 (IBM SPSS Statistics for Windows, Version 22.0, IBM Corporation, Armonk, New York, NY, USA).

## 3. Results

Patients with UC were older than their controls (40.0 ± 12.5 versus 37.7 ± 8.8 years, *p*-value = 0.014), with a lower BMI (25.3 ± 5.8 versus 27.4 ± 9.3 kg/m^2^; *p*-value = 0.007) and proportion of ever-smoking history (10.5% versus 20.5%; *p*-value = 0.004). Sex distribution did not differ significantly between UC patients and the controls (men: 59.6% versus 64.2%; *p*-value = 0.300) (Table 1).

Frequent intakes of coffee and tea were respectively associated with lower odds of UC in the unadjusted model, 0.59 (0.41, 0.85) and 0.50 (0.34, 0.72); the age- and sex-adjusted model, 0.56 (0.39, 0.81) and 0.48 (0.32, 0.71); and after further adjustment for BMI and smoking, 0.62 (0.42, 0.91) and 0.53 (0.35, 0.79). On the other hand, frequent intakes of carbonated soft drinks were associated with increased odds of UC in the unadjusted model, 5.28 (3.59, 7.77), age-and sex-adjusted model, 8.34 (5.33, 13.06), and after further adjustment for BMI and smoking, 9.82 (6.12, 15.76) (Table 2).

**Figure 1:**
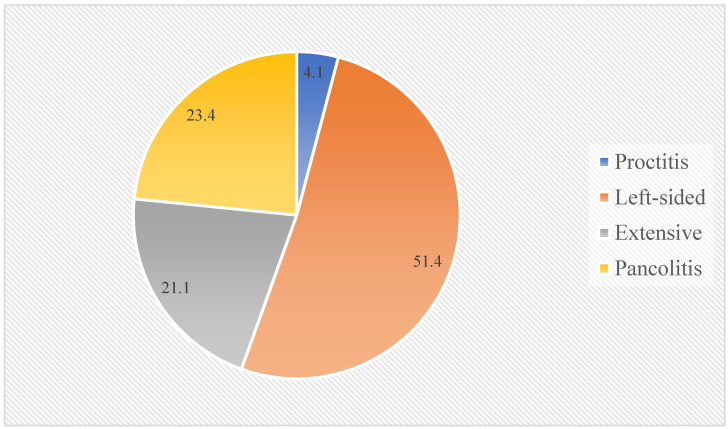
Extent of ulcerative colitis as detected by endoscopy.

Endoscopy showed that 23.4% of UC patients had pancolitis, 21.1% extensive, 51.4% left-sided, and 4.1% proctitis (Figure 1). Frequent intakes of coffee, tea, and carbonated soft drinks were not associated with the extent of UC in the multivariable-adjusted models (Table 3).

## 4. Discussion

To the best of our knowledge, this is the first study to investigate the association between beverage intakes and UC among an Arab population. This study indicated that frequent coffee and tea consumption could be associated with lower odds of UC, while frequent intake of carbonated soft drinks could be associated with higher odds of UC. The frequency of beverage consumption was not associated with UC extension.

Our results were in line with previous studies. A case-control study including populations from eight Pacific-Asian countries and Australia showed that daily coffee and tea consumption versus none could be protective against UC (0.49 (0.35, 0.69) and 0.63 (0.47, 0.85), respectively), while consuming soft drinks ≥ two times/week versus none tended to be associated with increased UC (1.55 (0.83, 2.90)) [11]. A population-based case-control study from Sweden showed that drinking ≥ three cups of coffee/day compared with none was associated with a lower prevalence of UC: 0.3 (0.2, 0.5) [8]. Another population-based case-control study from the Netherlands showed higher odds of UC among those who reported drinking Cola more than once/week compared with none: 1.6 (1.1, 2.3) [10]. A meta-analysis of 16 studies detected inverse associations between drinking coffee, 0.58 (0.33, 1.05), and tea, 0.69 (0.58, 0.83), with UC risk, while soft drink consumption was positively associated with UC risk, 1.69 (1.24, 2.30) [6].

Biologically, the protective effects of coffee and tea against UC risk are not unexpected, since oral caffeine administration was shown to ameliorate acute colitis via downregulating Chitinase 3-like 1 expression, a host protein known to facilitate bacterial attachment to intestinal epithelial cells to trigger UC [19]. Tea polyphenols enhanced antioxidant levels and attenuate inflammation in animal models with colitis [20]. On the other hand, sugar intake in a previous prospective study increased the risk of UC [9]. Sugar intake can alter gut microbiota associated with UC. Furthermore, carbonated soft drinks are usually associated with fast food and the Western diet, which are considered risk factors for UC [21].

Investigating the distribution of UC extension is beyond the aim of this study; however, we noticed that 44.5% of our UC patients presented with severe forms: 23.4% with pancolitis and 21.1% with extensive UC. This is higher than previous studies, which put the maximum prevalence of the extensive UC forms at 35% [22]. Such a finding should raise alarms about the delayed diagnosis of UC in Saudi Arabia, which could lead to worse outcomes and increased risk of intestinal surgeries [23].

Still, several limitations should be addressed. First, data about beverage consumption was collected via a self-administered questionnaire; therefore, reporting bias was likely. Second, we had no data about the subtypes of tea and coffee and whether the carbonated soft drinks were sweetened or not. Third, because of the limited number of cases per beverage consumption frequency group, we merged the groups representing non-daily beverage intake into one group. Thus, a dose-response association could not be assessed. Fourth, patients and their controls were recruited from one private polyclinic in Saudi Arabia. People who attend private clinics could have a higher socio-economic index and consequently consume more beverages, especially carbonated soft drinks, than other people who attend public clinics. Fifth, since controls were patients who had other gastrointestinal manifestations that could be related to beverage consumption, the association might be diluted. Sixth, UC patients and controls were collected over a relatively long period, during which the popular trends toward consuming beverages might have changed. Seventh, although UC patients were newly diagnosed, the high prevalence of extensive forms suggests that those patients had delayed UC diagnosis; therefore, their reports on beverage consumption might not have preceded the onset of UC. 

## 5. Conclusions

This manuscript showed that UC was negatively associated with frequent coffee and tea consumption but positively associated with frequent carbonated soft drink intake. A population-based prospective cohort study is needed to confirm our findings.

## Figures and Tables

**Table 1:** Comparison between ulcerative colitis patients and their controls.

Characteristics		Ulcerative Colitis	Controls	*p*-Value
Frequency		171	400	--
Age (mean ± Sd), years		40.0 ± 12.5	37.7 ± 8.8	0.014
Sex, %	Men	59.6	64.2	0.300
	Women	40.4	35.8	
Body mass index (mean ± Sd), kg/m^2^		25.3 ± 5.8	27.4 ± 9.3	0.007
Smoking, %		10.5	20.5	0.004

**Table 2:** Odds ratios and 95% confidence intervals for ulcerative colitis according to beverage consumption.

	Infrequent	Frequent
**Coffee**		
Ulcerative colitis, %	58.5	41.5
Controls, %	45.5	54.5
Model I	1	0.59 (0.41, 0.85)
Model II	1	0.56 (0.39, 0.81)
Model III	1	0.62 (0.42, 0.91)
**Tea**		
Ulcerative colitis, %	68.4	31.6
Controls, %	51.7	48.3
Model I	1	0.50 (0.34, 0.72)
Model II	1	0.48 (0.32, 0.71)
Model III	1	0.53 (0.35, 0.79)
**Carbonated soft drinks**		
Ulcerative colitis, %	36.8	63.2
Controls, %	75.5	24.5
Model I	1	5.28 (3.59, 7.77)
Model II	1	8.34 (5.33, 13.06)
Model III	1	9.82 (6.12, 15.76)

Model I: Unadjusted; Model II: Adjusted for age and sex; Model III: Adjusted for age, sex, body mass index, and history of smoking.

**Table 3:** Odds ratios and 95% confidence intervals for pancolitis according to beverage consumption.

	Infrequent	Frequent
**Coffee**		
Extensive, %	51.3	48.7
Mild to moderate, %	64.2	35.8
Model I	1	1.70 (0.92, 3.15)
Model II	1	1.31 (0.70, 2.45)
Model III	1	1.68 (0.88, 3.19)
**Tea**		
Extensive, %	59.2	40.8
Mild to moderate, %	75.8	24.2
Model I	1	2.16 (1.12, 4.15)
Model II	1	2.18 (1.09, 4.35)
Model III	1	2.00 (0.95, 4.19)
**Carbonated soft drinks**		
Extensive, %	32.9	67.1
Mild to moderate, %	40.0	60.0
Model I	1	1.36 (0.72, 2.56)
Model II	1	1.25 (0.62, 2.50)
Model III	1	1.09 (0.53, 2.25)

Model I: Unadjusted; Model II: Adjusted for age and sex; Model III: Adjusted for age, sex, body mass index, and history of smoking.

## Data Availability

Not applicable.
